# The Effects of Leptin Replacement on Neural Plasticity

**DOI:** 10.1155/2016/8528934

**Published:** 2016-01-03

**Authors:** Gilberto J. Paz-Filho

**Affiliations:** Department of Genome Sciences, The John Curtin School of Medical Research, The Australian National University, Canberra ACT 2612, Australia

## Abstract

Leptin, an adipokine synthesized and secreted mainly by the adipose tissue, has multiple effects on the regulation of food intake, energy expenditure, and metabolism. Its recently-approved analogue, metreleptin, has been evaluated in clinical trials for the treatment of patients with leptin deficiency due to mutations in the leptin gene, lipodystrophy syndromes, and hypothalamic amenorrhea. In such patients, leptin replacement therapy has led to changes in brain structure and function in intra- and extrahypothalamic areas, including the hippocampus. Furthermore, in one of those patients, improvements in neurocognitive development have been observed. In addition to this evidence linking leptin to neural plasticity and function, observational studies evaluating leptin-sufficient humans have also demonstrated direct correlation between blood leptin levels and brain volume and inverse associations between circulating leptin and risk for the development of dementia. This review summarizes the evidence in the literature on the role of leptin in neural plasticity (in leptin-deficient and in leptin-sufficient individuals) and its effects on synaptic activity, glutamate receptor trafficking, neuronal morphology, neuronal development and survival, and microglial function.

## 1. Introduction

Leptin is a 16-kDa hormone with cytokine-like actions (i.e., adipokine or adipocytokine), synthesized and secreted mainly by the white adipose tissue. As one of the most abundant adipokines, leptin has crucial effects on the regulation of food intake and energy balance [1]. Since its discovery in 1994, many additional actions have been described, with fundamental roles in lipid and glucose homeostasis, immunity, inflammation, bone physiology, reproduction, regulation of thyroid, growth hormone and adrenal axes, and tissue remodeling. Those actions have been identified mainly through animal models of leptin deficiency (namely, the* ob/ob* and the* db/db* mice), but also through studies carried out in humans with leptin deficiency: patients with lipodystrophy syndromes, hypothalamic amenorrhea, and congenital leptin deficiency (CLD) due to mutations in the leptin gene [2].

Humans with leptin deficiency develop metabolic dysfunctions such as increased insulin resistance, hyperglycemia, dyslipidemia, endocrine disruptions, and fatty liver disease. In addition, morbid obesity, impaired cognitive development, and potentially lethal T-cell hyporesponsiveness have been reported in patients with mutations in the leptin gene [3].

More recently, animal and human studies have shown that leptin has remarkable effects on neural plasticity and cognition [4]. In humans with leptin deficiency, leptin replacement therapy (LRT) has led to time-dependent increases in gray matter concentration in diverse brain regions (including the hippocampus) and to changes in activation of regions traditionally linked to hunger, satiety, and reward. Moreover, improvements in neurocognition have been reported in isolated cases [5]. In normoleptinemic humans, circulating leptin levels have also been associated with alterations in cognition [6]. Furthermore, studies suggest that leptin is neuroprotective [7], and low leptin levels may play a role in the pathogenesis of neurodegenerative diseases such as Alzheimer's disease (AD) and Parkinson's disease (PD) [8].

In this paper, the effects of leptin on the structure and function of the central nervous system will be described. The literature presenting the association (or lack therefore) between leptin and AD will be reviewed, and the future directions for studies on the role of leptin replacement in neural plasticity will be discussed.

## 2. Biology of Leptin

Leptin has structural homology with long-chain helical cytokines including IL-6, IL-11, IL-12, and oncostatin M. Leptin has tridimensional characteristics of a four-helical bundled cytokine, similar to those cytokines that activate the Janus kinase (JAK) and Signal Transducer and Activator of Transcription (STAT) pathway [9]. In fact, the structural similarities that leptin shares with the IL-6 cytokine family led to functional* in vitro* signaling studies, which proved that leptin does in fact activate the JAK2-STAT3 pathway, although STAT1, STAT5, and STAT6 may be activated by leptin as well [10].

Its receptor, Ob-R (or Lep-R), is structurally similar to members of the class I cytokine receptor (gp130) superfamily. There are at least six different isoforms of the leptin receptor in rat: Ob-Ra, Ob-Rb, Ob-Rc, Ob-Rd, Ob-Re, and Ob-Rf; all of them are products of six alternatively spliced forms of the Ob-R gene [11]. Ob-Rb is the only long form, containing a long cytoplasmic region with several motifs required for signal transduction and activation of the intracellular pathways. There are four truncated (short) forms (Ob-Ra, Ob-Rc, Ob-Rd, and Ob-Rf); Ob-Ra is regarded as a leptin transporter across the blood-brain barrier (BBB) and a leptin degrader, and Ob-Re is a secreted form lacking intracellular and transmembrane domains, serving as plasmatic leptin-binding protein. All isoforms (except Ob-Rb) share the same identical extracellular ligand-binding domains, differing at the C terminus. All these isoforms are involved in the mediation of leptin's actions in peripheral organs and in the brain.

The murine and human leptin receptors are highly similar in amino acid sequences for both the extracellular (78% identity) and intracellular domains (71% identity) [12]. In humans, a soluble isoform (sOb-R, homologous to murine Ob-Re) and four different membrane-anchored Ob-R isoforms have been described: Ob-R219.2, homologous to murine Ob-Ra; Ob-Rfl, homologous to murine Ob-Rb; Ob-R219.3, homologous to murine Ob-Rc; and Ob-R219.1, homologous to murine Ob-Rd [13]. No Ob-Re transcript has been found in humans: in rodents, to create Ob-Re, the splice site at the 3′-end of exon 14 is skipped, leading to the transcription of a stop codon and a polyadenylation signal; in humans, the sequence 5′ of exon 14 does not have a polyadenylation signal. Instead, Ob-Re is generated by proteolytic cleavage. Ob-Rf has only been found in rat.

Besides activating the JAK2/STAT pathway, leptin controls other key signaling pathways: mitogen-activated protein kinase (MAPK)/extracellular signal-regulated kinase- (ERK) 1/2, and phosphatidylinositol 3 kinase (PI3K)/protein kinase B (Akt)/mammalian target of rapamycin (mTOR)/forkhead box protein O1 (FoxO1) pathways [24]. The activation of MAPK/ERK pathway is believed to be the main mechanism involved in the regulation of cell cycle and proliferation [25]; the PI3K pathway is an acute phase contributor of inflammation and is also involved in the phosphorylation of insulin receptor substrate (IRS), important for glucose homeostasis. Leptin's intracellular pathways are downregulated by proteins such as suppressor of cytokine signaling 3 (SOCS3), protein tyrosine phosphatase 1B (PTP1B), and src homology-2-containing protein tyrosine phosphatase 2 (SHP2). The upregulation of genes synthetizing such proteins is the main contributor to leptin resistance [26].

In order to elicit central actions, leptin must cross the BBB through a saturable, passive transport across the barrier. The leptin receptor isoforms Ob-Ra and Ob-Rc have been shown to mediate BBB transport of leptin, and dysfunctional receptors may lead to leptin resistance. More recently, it has been shown that the tanycytes in the median eminence take up blood-borne leptin in an Ob-Rb dependent manner, constituting a route for entry into the hypothalamus [27]. Hypertriglyceridemia may also be another contributor to leptin resistance, as high triglyceride levels decrease leptin transport across the BBB [28].

An important function of leptin is to regulate energy expenditure and food intake, by its actions in the arcuate nucleus (ARC) of the hypothalamus. In this area, leptin binds to its receptors, expressed in two different neuronal populations: the ones that express agouti-related peptide (AgRP) and neuropeptide Y (NPY) and those that express the peptide cocaine and amphetamine-related transcript (CART) and the peptide pro-opiomelanocortin (POMC). Leptin exerts anorexigenic effects by inhibiting the AgRP/NPY neurons and by stimulating the POMC/CART neurons [1].

## 3. Leptin Replacement Therapy

Studies evaluating LRT in patients with leptin deficiency due to mutations in the* LEP* gene allow the understanding of the physiological effects of its replacement in leptin-sensitive humans not previously exposed to the endogenous adipokine. In humans, leptin deficiency is observed in cases of patients with lipodystrophy syndromes, hypothalamic amenorrhea, and congenital leptin deficiency (CLD) due to mutations in the leptin gene [2]. Several trials of LRT in such patients have been reported in the literature, mostly evaluating the metabolic and endocrine effects of LRT. However, some studies have evaluated the central effects of LRT.

The current presentation of leptin that is available for human therapy is recombinant methionyl human leptin (metreleptin, Myalept, Aegerion Pharmaceuticals, Inc.). In the US, Myalept is available only through the Myalept Risk Evaluation and Mitigation Strategy (REMS) Program, under which prescribers must be certified with the program by enrolling in and completing training. Metreleptin is composed by the 146 amino acids of the mature form of human leptin, with the addition of a methionyl residue at the N-terminal end. Myalept has been recently approved by the FDA for the treatment of congenital or acquired generalized lipodystrophy (non-HIV-related), but it has also been trialed in patients with the partial forms of the disease (approval depending on results from more trials evaluating safety and effectiveness). The recommended starting dose varies by gender and body weight, to a maximum daily dose of 0.13 mg/kg/day (if body weight ≤ 40 kg) and 10 mg/day (if body weight > 40 kg). Metreleptin is preferably administered once daily at the same time every day, subcutaneously. It is eliminated unmetabolized via renal clearance, and its most common reported adverse reactions (≥10%) include headache, hypoglycemia, decreased weight, and abdominal pain. Autoimmune disorder progression (autoimmune hepatitis and membranoproliferative glomerulonephritis) and T-cell lymphoma have been reported in patients with lipodystrophy being treated with metreleptin [29]. Also, hypersensitivity reactions (e.g., urticaria or generalized rash) have been reported [30]. It is contraindicated for patients with general obesity not associated with congenital leptin deficiency and hypersensitivity to metreleptin. Its safety in pregnant women is unknown. In nursing women, either metreleptin therapy or nursing should be discontinued.

Anti-metreleptin antibodies have been identified in nearly all lipodystrophy patients (>95%) treated with metreleptin from two NIH studies (NIH Studies 991265 and 20010769) and Study FHA101 (sponsor-initiated) [30]. The effects of those antibodies have not been well characterized yet, but they can have neutralizing activity and lead to loss of metabolic control by inhibiting leptin action [31, 32].

## 4. Effects of Leptin Replacement Therapy in the Brain of Patients with Mutations in the Leptin Gene

Cases of CLD caused by mutations in the leptin gene are rare: to date, a total of 38 individuals of Turkish (*n* = 6) [5, 33–35], Pakistani (*n* = 27) [31, 36–40], Austrian (*n* = 1) [41], Egyptian (*n* = 2) [14], Russian (*n* = 1) [42], and Indian (*n* = 1) [43] origins, harboring different mutations in the leptin gene, have been reported. Physiological doses of metreleptin have been evaluated in the treatment of CLD in patients of Turkish, Pakistani, and Austrian background, with remarkable effects on body composition, metabolism, endocrine and immune systems, brain, and behavior. Since leptin plays crucial roles in the immune system, many untreated affected individuals die during childhood due to infections and sepsis. In those cases, leptin therapy may be lifesaving [44].

For patients with CLD, the usual initial dose of metreleptin is 0.02–0.04 mg/kg/day, calculated to achieve 10% of predicted serum leptin concentration (based on pharmacodynamic and pharmacokinetic data from AMGEN Inc., Thousand Oaks, California, USA). Dose remains the same if weight reduces or stabilizes. If weight increases over two consecutive months, the dose is increased to achieve 20%, and subsequently 50%, 100%, and 150% predicted serum leptin concentrations. Dose can be reduced if excessive weight loss occurs [2].

Studies on these patients while on and off LRT have shown that its effects in brain plasticity are remarkable. In Turkish adults with a Arg105Trp missense mutation, 18 months of LRT led to relative gray matter concentration increases in areas associated with regulation of hunger or motor control in human subjects, namely, the frontal cortex (primarily in the left anterior cingulate gyrus), left inferior parietal lobule, and left cerebellum [45]. Peak or maximum effect within significant clusters of voxels were observed in the left anterior cingulate gyrus, left inferior parietal lobule, and left cerebellum, at the same stereotaxic coordinates in scans acquired a year before (after 6 months of LRT), with fewer voxels at 18 months. No significant effect occurred in the hypothalamic region, where leptin determines its effects on food intake. Annual withholding of replacement for 11–36 days per year over 3 years (mean 28.6 days/year) reversed this effect in the left anterior cingulate gyrus and cerebellum with larger extent, and in the left inferior parietal lobule (not significant). Furthermore, short-term treatment reinitiation for 11–22 days/year (mean 16.1 days/year) did not lead to recovery of gray matter concentration in the three expected locations but caused an unexpected increase in gray matter concentration in the posterior half of the left thalamus, particularly the pulvinar nuclei, which are areas implicated in neural circuits regulating food-seeking through relay of taste [15]. The gray matter changes in the cerebellum were directly caused by leptin, whereas the changes in the anterior cingulate gyrus and in the inferior parietal lobule were explained by its effects on body mass index (BMI). Furthermore, an unexpected negative correlation between BMI and gray matter structure in the right inferior temporal gyrus was observed, and a positive correlation between duration of leptin supplementation and gray matter structure in the right hippocampus was also observed [15], in concordance with previous studies showing that leptin levels are positively correlated with hippocampal structure and function [46], which may be related to leptin's effects on memory.

In functional magnetic resonance imaging (MRI) studies, leptin replacement reduced activation of regions linked to hunger (insula, parietal, and temporal cortex) and enhanced activation of regions linked to inhibition and satiety (prefrontal cortex, mainly middle, superior, and medial frontal gyri, and regions linked to satiety and inhibition of high-calorie food intake) [16], as well as the posterior lobe of the cerebellum, the brain region with the highest concentration of leptin receptors [17]. When LRT was withheld, activity was greater in the insula (the primary gustatory cortex) and other temporal/parietal regions (involved in the sensation of hunger), as well as occipital and limbic regions. These results show that leptin has extrahypothalamic effects in the regulation of food intake, reversibly altering neural structure and function and modulating plasticity-dependent brain physiology in response to food cues. Although the cerebellum is not traditionally regarded as an area responsible for the control of food intake, it may be involved in the suppression of motivational food-seeking, when leptin-sufficient individuals are not as hungry. Moreover, in positron emission tomography studies, we have shown that resumption of leptin treatment, after long-term replacement and short-term removal, does not significantly increase striatal D2/D3 receptor availability [21].

In a 15-year-old female Austrian patient, acute leptin therapy did not reduce activity in the hunger-related regions we have previously reported. In functional MRI studies, after 3 days of LRT, Frank et al. observed the activation of reward- and food-processing areas (ventral striatum and the orbitofrontal cortex, resp.). Acute and long-term (6 months of LRT) activation differences were observed in the amygdala and substantia nigra/ventral tegmental area (both decreased) and in the orbitofrontal cortex (increased). When comparing responses to pictures showing high-calorie versus low-calorie food, the authors observed that the activation of the hypothalamus to high-calorie pictures was decreased over time, whereas low-calorie stimuli led to increased activation of the hypothalamus [18]. In their 1- and 2-year follow-up study, the long-term effects in the amygdala and in the orbitofrontal cortex were sustained, with a decrease of the frontopolar cortex activity. Long-term effects in the hypothalamus showed an assimilating pattern for the response to high- versus low-calorie food, and hedonic regions showed only acute effects after 3 days of LRT [19].

Functional MRI studies carried out in patients of Pakistani background showed that the leptin-deficient state was associated with activation in the anteromedial ventral striatum (nucleus accumbens and caudate nucleus) and posterolateral ventral striatum (putamen and globus pallidus) and 7 days of LRT reduced activation in the nucleus accumbens-caudate and putamen-globus pallidus regions [20].

The procognitive effects of leptin were observed in the youngest Turkish male patient, who showed improvements of several subtests within neuropsychological functioning tests [5]. Neurocognitive assessments (Differential Ability Scales, a measure of general verbal and nonverbal functioning; and selected subtests from the NEPSY, a measure of neuropsychological functioning in children) were conducted at ages 5 (leptin-naïve state), 6, and 7 (on LRT). The patient's pretreatment Differential Ability Scales (DAS) verbal, nonverbal, preacademic, and short-term memory cluster scores were lower than the scores for age-matched controls. However, LRT was followed by an upward trend in development, with scores generally normalizing at age 7. Similar upward trend was observed for scores in the NEPSY visual-spatial and language-memory subtests [5].

## 5. Effects of Leptin Replacement Therapy in the Brain of Patients with Lipodystrophy

Patients with lipodystrophy share some of the clinical manifestations of CLD, due to low/absent blood leptin levels caused by the generalized or partial absence of subcutaneous adipose tissue. A recent meta-analysis has shown that LRT decreases fasting glucose, HbA1c, triglycerides, total cholesterol, liver volume, and aspartate aminotransferase levels in patients with LS not associated with highly active antiretroviral therapy use [47]. Unfortunately, no structural studies have been conducted in lipodystrophy patients; the only functional study in lipodystrophy patients showed that LRT increased food-related neural activity in the orbitofrontal cortex and suppressed activity in the amygdala, hippocampus, insula, caudate, and putamen, under postprandial conditions (which has little involvement in the regulation of neural activity while fasting) [22].

## 6. Effects of Leptin Replacement Therapy in Patients with Acquired Leptin Deficiency

The structural and functional effects of LRT have also been evaluated in three women with acquired leptin deficiency (defined as hypoleptinemia for at least 6 months, coincident with strenuous exercise and/or low body weight, without any significant comorbid medical conditions, including eating disorders). In hypoleptinemic women, brain structure and response to visual stimuli while fasting were similar to those of normal controls. In the fed state, participants showed increased activation in precuneus, insula, and dorsolateral prefrontal cortices and decreased activation in insula in response to viewing food. After 1 week of LRT, fasting hypoleptinemic women showed increased activation in bilateral insula, dorsolateral prefrontal, and medial frontal cortices in response to viewing food. In the fed state, they showed less activation in the precuneus, middle frontal, thalamic, insular, and parahippocampal cortices. After 24 weeks of LRT, while fasting, hypoleptinemic women showed greater activation in insular and inferior frontal cortices in response to viewing food. After feeding, they showed less activation in midbrain, cuneus, midcingulate, bilateral parietal, and superior prefrontal cortices. In this study, the authors showed that hypothalamic activity is modulated by LRT, which decreases functional connectivity of the hypothalamus to feeding-related areas. Despite having identified changes in brain function after LRT, this study has not shown any changes in brain structure, which the authors attribute to the fact that their patients had normal brain development during their early life leptin-sufficient period and had structurally normal brains [23]. The most relevant changes brain changes after LRT are summarized in Table 1.

## 7. Physiology of Leptin in the Brain

At the molecular level, leptin stimulates neurogenesis, axon growth, synaptogenesis, and dendritic morphology, both pre- and postnatal life [48]. Leptin also has neuroprotective actions, by inhibiting apoptotic cell death and improving cell survival through the regulation of apoptotic enzymes (by inhibiting the expression of Bcl-xL, caspases and TRAIL ligand, and activating the synthesis of neurotrophic factors such as BDNF), protecting against glutamatergic cytotoxicity, protecting against oxidative stress via expression of the membrane antioxidant MnSOD and stabilization of mitochondrial membranes, and promoting the proliferation of hippocampal progenitor cells [49–52]. Leptin regulates the synapse morphology of hippocampal neurons, enhancing the motility and density of dendritic filopodia [53]. Also, leptin regulates the development of oligodendroglial cells [54], which may contribute to the structural changes in gray matter. Furthermore, leptin affects neuron excitability and synaptic transmission, via the activation and trafficking of ATP-sensitive K^+^ channels (in the hypothalamus) and Ca^2+^-activated K^+^ channels (in the hippocampus) [55, 56]. By enhancing NMDA receptor function, leptin facilitates the conversion of short-term potentiation into long-term potentiation, rapidly remodeling dendrites and facilitating spatial learning and memory performance in mice [55, 57–59]. Furthermore, leptin counteracts glucocorticoids' inhibitory effect on hippocampal neurogenesis [60].

## 8. Leptin in Neurocognitive Disorders

Given the fact that leptin plays important roles in brain development and activity, several studies evaluated its effects on brain size and function. In healthy individuals, circulating leptin levels have been inversely correlated with total and regional brain volumes, independent of body size [45, 61, 62]. Besides further confirming the correlation between leptin levels and brain size, a prospective study of 785 healthy persons from the Framingham cohort showed that higher leptin levels were associated with a lower risk of dementia and AD in lean, leptin-sensitive people [63]. Participants in the lowest leptin quartile were at a 4-fold higher risk for developing AD in 12 years, compared to the participants in the highest quartile (25% versus 6%). The lack of association between leptin and risk of dementia was not seen in obese individuals, possibly due to leptin resistance [64]. Similar studies have also associated low leptin levels with cognitive impairment, particularly AD [65–68], but others have not identified such correlation [69, 70]. It is still unclear whether low leptin levels are involved in the pathogenesis of AD, either by directly altering neuronal and microglial physiology, by predisposing to the accumulation of toxic amyloid beta and neurofibrillary tangles [71], or by increasing central insulin resistance and inflammation [72–74]. Also, not only absolute leptin levels, but also leptin resistance may be involved in the pathogenesis of AD, as demonstrated in a study where leptin signaling was altered in the hippocampus of AD patients [75]. In transgenic mice models of AD, LRT promotes significant cognitive improvements [57]. Although leptin is a promising AD therapeutic target [76, 77], LRT clinical trials on AD patients have not been conducted so far.

## 9. Conclusions

More than twenty years after its discovery, leptin is now regarded as more than a regulator of food intake and energy expenditure. Animal and human models of leptin-deficiency have demonstrated that leptin is an adipokine that acts in the central nervous system and alters synaptic activity, glutamate receptor trafficking, neuronal morphology, development and survival, and microglial function. Furthermore, leptin replacement therapy in such models has elicited neuroplastic and functional changes, which translated into the activation of specific regulatory brain areas and normalization of behavioral responses to food stimuli. Interestingly, leptin replacement altered gray matter concentration of areas not related to hunger and satiety, such as the hippocampus—a brain area traditionally linked to memory and cognition. In one leptin-deficient human, treatment improved several cognitive parameters, indicating that leptin's effects in the brain may lead to clinically relevant changes. However, those changes, particularly regarding brain volume, were not evident in humans with acquired leptin-deficiency, whose brain developed normally while these patients were leptin-sufficient early in their lives. This suggests that the neuroplastic effects of leptin in leptin-sufficient individuals may not be broad enough to be detected by brain imaging, or that leptin has no neuroplastic effects at all in previously leptin-sufficient humans.

Furthermore, observational studies have shown that leptin plays a role in cognition and in the pathogenesis of Alzheimer's disease. More specifically, lower leptin levels may increase the risk for the development of Alzheimer's due to a decrease in leptin's functional and structural beneficial effects to the central nervous system. However, results are conflicting, and confounding factors must be taken into account, such as body composition, arterial blood pressure, insulin resistance, and central inflammation.

It is unquestionable that leptin plays a role in neuroplasticity, at least in models of early-life leptin deficiency. Future studies need to establish whether a decrease in leptin's actions can lead to neurodegenerative disorders and whether the neuroplastic effects of leptin are also present in leptin-sufficient individuals. If so, clinical trials need to evaluate whether leptin replacement therapy is safe and effective for the prevention or treatment of such diseases.

## Figures and Tables

**Table 1:** Effects of leptin replacement therapy on neural plasticity, neural function, and cognition.

	Leptin deficiency due to mutations in the leptin gene	Lipodystrophy	Acquired leptin deficiency
Brain structure	Increases in gray matter concentration in areas associated with regulation of hunger, motor control [14], relay of taste, and hippocampus [15].	Not evaluated.	No changes.

Brain function	Reduced activation of regions linked to hunger and enhanced activation of regions linked to inhibition and satiety, as well as cerebellum [16, 17]. Activation of reward- and food-processing areas [18], with sustained activation effect in the amygdala and in the orbitofrontal cortex, and decrease of the frontopolar cortex activity [19]. Activation in the anteromedial and posterolateral ventral striatum; reduced activation in the nucleus accumbens-caudate and putamen-globus pallidus regions [20]. No change in striatal D2/D3 receptor availability [21].	Increased food-related activity in the orbitofrontal cortex; decreased activity in the amygdala, hippocampus, insula, caudate, and putamen, under postprandial conditions [22].	Acute effects: Increased activation in bilateral insula, dorsolateral prefrontal, and medial frontal cortices in response to viewing food (while fasting). Less activation in the precuneus, middle frontal, thalamic, insular, and parahippocampal cortices (in the fed state). Chronic effects: Increased activation in insular and inferior frontal cortices in response to viewing food (while fasting). Decreased activation in midbrain, cuneus, midcingulate, bilateral parietal, and superior prefrontal cortices (after feeding) [23].

Cognition	Improvements of several subtests within neuropsychological functioning tests [5].	Not evaluated.	Not evaluated.
